# Simple tests of cardiorespiratory fitness in a pediatric population

**DOI:** 10.1371/journal.pone.0238863

**Published:** 2020-09-04

**Authors:** Brittany S. Bruggeman, Heather K. Vincent, Xiaofei Chi, Stephanie L. Filipp, Rebeccah Mercado, François Modave, Yi Guo, Matthew J. Gurka, Angelina Bernier

**Affiliations:** 1 Division of Endocrinology, Department of Pediatrics, University of Florida College of Medicine, Gainesville, FL, United States of America; 2 Division of Research, Department of Orthopedics and Rehabilitation, University of Florida College of Medicine, Gainesville, FL, United States of America; 3 Department of Health Outcomes and Biomedical Informatics, University of Florida College of Medicine, Gainesville, FL, United States of America; 4 Department of Pediatrics, University of Florida College of Medicine, Gainesville, FL, United States of America; Universidade Federal de Juiz de Fora, BRAZIL

## Abstract

A progressive, treadmill-based VO_2max_ is the gold standard of cardiorespiratory fitness determination but is rarely used in pediatric clinics due to time requirements and cost. Simpler and shorter fitness tests such as the Squat Test or Step Test may be feasible and clinically useful alternatives. However, performance comparisons of these tests to treadmill VO_2max_ tests are lacking. The primary aim of this cross-sectional study was to assess the correlation between Squat and Step Test scores and VO_2max_ in a pediatric population. As secondary outcomes, we calculated correlations between Rated Perceived Exertion Scale (RPE) scores, NIH PROMIS Physical Activity scores, and BMI z-score with VO_2max_, and we also evaluated the ability of each fitness test to discriminate low and high-risk patients based on the FITNESSGram. Forty children aged 10–17 completed these simple cardiorespiratory fitness tests. Statistically significant correlations were observed between VO_2max_ and the Step Test (r = -0.549) and Squat Test (r = -0.429) scores, as well as participant BMI z-score (r = -0.458). RPE and PROMIS scores were not observed to be correlated with VO_2max_. Area Under the Receiver Operator Curve was relatively high for BMI z-scores and the Step Test (AUC = 0.813, 0.713 respectively), and lower for the Squat Test (AUC = 0.610) in discriminating risk according to FITNESSGram Scores. In this sample, the Step Test performed best overall. These tests were safe, feasible, and may add great value in assessing cardiorespiratory fitness in a clinical setting.

## Introduction

Poor cardiorespiratory fitness (CRF) is the fourth-leading risk factor for cardiovascular disease and is a stronger predictor of mortality than hypertension, obesity, hyperlipidemia, or type 2 diabetes mellitus in a number of studies [1–4]. In the pediatric population, higher levels of CRF are associated with a healthier metabolic and cardiovascular profile [5, 6] and have been linked to improved mental health and academic performance [7, 8]. The addition of CRF to traditional risk factors significantly improves prediction of adverse health outcomes [9]. Despite these observations, while other predictors of cardiovascular outcomes such as hemoglobin A1c, lipids, body mass index (BMI), and blood pressure are routinely monitored in pediatric clinics, CRF is not.

A comprehensive progressive treadmill test that determines the maximal rate of oxygen use (VO_2max_) is the gold standard of CRF determination [10, 11], but is rarely used in clinics due to time requirement, space needs and cost [3]. Testing requires up to 1.5 hours, an electrocardiogram, blood pressure monitoring, and volume of expired O_2_/CO_2_ collected through a mask worn during the test. The limited use of fitness testing in clinic is unfortunate as the VO_2max_ values can be clinically used to discriminate whether children are at high risk for metabolic or cardiovascular morbidity and detect related cardiac arrhythmias or pulmonary issues. Moreover, the test results are used to formulate exercise prescriptions and inform the child of what exercise should feel like when starting a lifestyle change program. The treadmill test directly captures real-time cardiovascular, metabolic, pulmonary and perceived effort responses and generates a VO_2max_ value which can be compared to national FITNESSgram norms [12]. Test results are related to patient characteristics including BMI and general level of physical activity, where sedentary individuals with high adiposity have low VO_2max_ values.

Simple and short fitness tests such as the 45-second Squat Test [13] or the 3-minute Step Test [14] may be useful alternatives in the clinical setting, as they require much less time, space, and staffing to complete and can be performed in a primary care or metabolic clinic. These tests estimate general fitness categories (‘poor’ to ‘excellent’) or VO_2max_ values using heart rate (HR) responses. In the Squat Test, a participant completes 30 squats in a 45-second time frame. The 3-minute Step Test involves a participant stepping up and down on a 12-inch step for three minutes at an established cadence [14]. Heart rates are monitored prior to the tests, immediately after, and one minute post. The Squat Test is scored using the Ruffier and Dickson indices [13]. Several scoring mechanisms have been proposed for the Step Test in children, including simply measuring the one minute post-test heart rate [14] and a predictive formula created by Jacks et al [15]. While these two tests do not assess the full complement of physiological responses like the progressive treadmill test, these tests can provide children, families and clinicians with a starting point of discussion about health risks related to fitness and engagement in exercise.

Incorporation of these tests into clinical practice first requires comparison of performance to the gold standard treadmill test. Presently it is unclear how closely the Squat or the Step Test perform relative to the VO_2max_ treadmill test in children and whether the test scores are also related to BMI or physical activity levels. Therefore, the purpose of this study was to determine the association between fitness scores from the VO_2max_ treadmill test, Squat Test and Step Test and how well each test could discriminate low and high risk patients using FITNESSGram standards. We hypothesized that the Squat and Step Tests would be correlated with the VO_2max_ in this pediatric sample, and that all three fitness determinations would be able to effectively discriminate low and high risk populations as defined by FITNESSGram standards [12].

## Methods

### Study design

This was a cross-sectional comparative study of three fitness tests in apparently healthy children. This study and all procedures was approved by the University of Florida (UF) Institutional Review Board (201602270).

### Participants

Children aged 10–17 were recruited (N = 40) from the Gainesville, Florida area and surrounding community. Forty participants were included as this sample size was sufficient to achieve stability in statistical models based on analysis of the Root Mean Square Error (RMSE) in a similar study of adults performed at our institution [16]. Flyers were posted in campus buildings, schools, public locations, and UF Pediatric Clinics for recruitment. Exclusion criteria were physical activity limitations, presence of heart conditions, chest pain or dizziness with exercise, uncontrolled asthma, uncontrolled diabetes, recent musculoskeletal injury, or other serious illnesses. All children and parents completed a written informed assent and consent, respectively.

### Procedures

**Fig 1:**
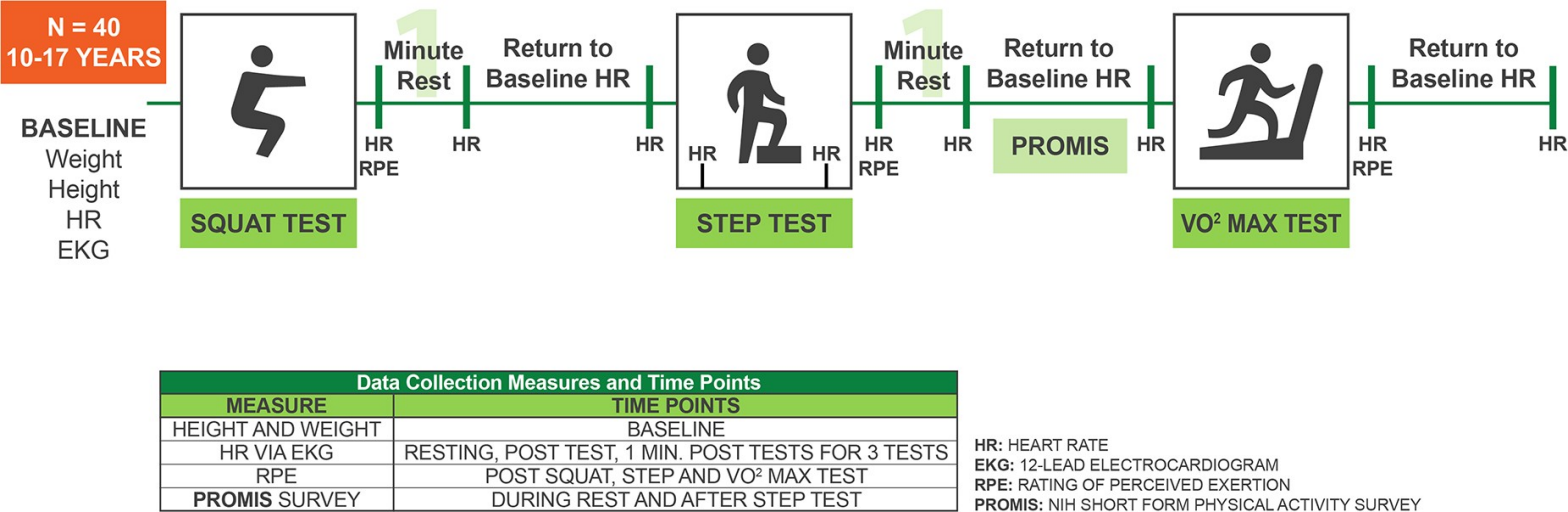
Study design schematic for cardiorespiratory fitness testing. Participants’ baseline height, weight, body mass index (BMI), heart rate (HR), and electrocardiogram (EKG) output were first measured. They then completed the Squat Test; HR and rating of perceived exertion scale (RPE) measures were performed; and a one-minute post-test HR was measured. HR was allowed to return to baseline before proceeding to the Step Test; where HR was measured prior to the test, at one and two minutes during the test and immediately after the test (along with RPE measures), and after one minute of rest. HR was then allowed to return to baseline while the NIH PROMIS Short Form Physical Activity Survey was completed. HR was measured prior to VO2max treadmill testing and throughout the test, RPE scores were measured throughout the test and immediately after testing, and HR was allowed to return to baseline, which signaled the end of the testing procedure.

Testing was performed on one day in the UF Health Sports Performance Center setting, with the total time allotment not exceeding two hours. Prior to testing, height and weight were collected using a medical grade scale and BMI was calculated. Tests were performed in the following order, with the heart rate returning to baseline prior to moving on to the next test: 1) Squat Test; 2) Step Test; 3) completion of the NIH PROMIS Short Form Physical Activity Survey; and 4) VO_2max_ treadmill test (**Fig 1)**.

#### Squat test

A 12-lead electrocardiogram (Quinton Q Stress; Cardiac Science Corporation, Bothell WA, USA) was placed on the participant. The coordinator then instructed the participant to sit and rest for five minutes. Resting HR and electrocardiogram output was then collected (P0). The participant was then asked to complete 30 squats in 45 seconds, paced by a metronome and counted out loud by the coordinator. The squatting required bending the knees to approximately a 90 degree angle, while keeping the back straight and the arms extended straight forward. Immediately after the squats were completed, HR was collected again (P1). The rating of perceived exertion (RPE) was measured at this time using an 11-point categorical RPE scale, where 0 = no exertion at all and 10 = maximal exertion possible [17]. The participant sat down for one minute of recovery, and the final HR was collected at this time (P2). The Ruffier and Dickson indices were then calculated [13] to score the test:

**Ruffier index:** ((P0+P1+P2)-200)/10
**Dickson index:** ((P1-70)+2(P2-P0))/10

#### Step test

Once the participant’s HR returned to resting pre-test levels, the Step Test was then performed [14]. A pre-exercise HR and electrocardiogram were first captured in a seated position (T0). The coordinator provided standardized instructions to each participant and demonstrated the stepping process on a 12-inch step. A metronome was set at an established cadence of 96 steps per minute at a rhythm of “up, up” and “down, down.” HR and 12-lead electrocardiogram outputs were collected at minute one (T1), minute two (T2), minute three (T3), and after being seated for recovery for one minute (T4). A score based upon a predictive formula created by Jacks et al [15] was calculated to predict VO_2max._

**Jacks et al formula:** -2.045 + (height in inch*0.062) + 100*[1/3*(T1+T2+T3)/T0]*(-0.411) + (T0*0.011)

We also applied the Ruffier index formula and Dickson index formula to the Step Test to assess if these formulas would demonstrate a better correlation compared to the one minute post-test heart rate alone.

**Ruffier index for Step Test:** ((T0+T3+T4)-200)/10
**Dickson index for Step Test:** ((T3-70)+2(T4-T0))/10

#### NIH PROMIS short form physical activity survey

Participants then completed the eight-question pediatric version of the PROMIS survey [18] regarding their physical activity over the past seven days. This survey was chosen to compare participants’ reported activity to measured fitness.

#### Treadmill VO2max test

Once HR values had returned to resting, pre-exercise values, the coordinator provided the standardized instructions for the treadmill test. A modified Bruce treadmill protocol was selected because the test commences at a lower workload than the standard Bruce Protocol [19–23] and helped children who were unaccustomed to treadmills feel more comfortable with the process. Stages were three minutes long. The first three stages were at 1.7 mph with 5% grade changes per stage. From stage four on, the grade changed by 2% every three minutes. The speed increased from 2.5 mph at stage four, to 3.4 mph, 4.2 mph, 5.0 mph, 5.5 mph to 6.0 mph. Prior to every test, the gas chamber system was calibrated using standardized gas mixtures and a volume calibration was performed using a 3L syringe. The child was fitted with a rubberized mask that was connected to the metabolic gas analyzer (Viasys, CareFusion; Yorba Linda, CA; USA). During the test breath-by-breath gas exchange, ventilation patterns, HR and heart electrical activity were continuously collected. RPE was used as a subjective measure of participant level of effort at the end of each stage of the test using the 0–10 point RPE scale. Dyspnea and angina were monitored at the end of each stage using the four level scales provided in the American Heart Association statement for clinical exercise testing [24]. A true maximal test was achieved if the participant attained the guidelines established by the American College of Sports Medicine: achieving a plateau in VO_2_ values despite an increase in work rate, achieving a HR value of 85% of its age predicted value, or achieving a respiratory exchange ratio of ≥1.15 and an RPE approaching maximum effort [17]. Due to the non-linear relationship between oxygen consumption and body mass, the VO_2_ values were allometrically scaled to prevent errors in metabolic calculations in persons with higher body weight. VO_2_ values were raised to a recommended exponent of 0.75 [25].

#### BMI z-score

We used the Center for Disease Control and Prevention SAS program to calculate BMI z-score based on the child’s sex and age in months based upon the U.S. CDC 2000 growth charts for children 24–239 months [26].

#### Feasibility and safety

The feasibility of tests was determined by ease of execution by the staff and relatively low participant burden. Safety was tracked by adverse events related to the tests, and included but was not limited to cardiovascular adverse events or responses, joint pain or any tripping or falling on equipment.

### Data processing and statistics

We hypothesized that the Squat and Step Tests would be normally distributed and have strong correlation with the VO_2max_ in this patient population. As secondary outcomes, we compared Rated Perceived Exertion Scale (RPE) scores, PROMIS scores, and BMI z-score with VO_2max_ and evaluated the discrimination of each fitness determination as compared to FITNESSGram scores, which classifies children into high and low risk fitness categories based upon whether VO_2max_ scores meet FITNESSGram standards [12]. We hypothesized that there would be a stronger correlation between the VO_2max_ and the PROMIS measure than the Squat and Step tests, and that both RPE score and BMI z-score would correlate with VO_2max_. We hypothesized that all three fitness determinations and BMI z-score would be able to effectively discriminate low and high risk populations as defined by FITNESSGram standards [12]. Distribution of scores for each test was evaluated using a normal probability plot and histogram.

#### Correlation among tests and performance

Pearson’s r correlations were calculated among the Squat Test, Step Test, and VO_2max_.

#### Discriminative ability of tests to find risk in children using FITNESSGRAM

Receiver operating characteristic (ROC) area under the curve (AUC) values were calculated to assess the ability to discriminate high and low risk as classified by FITNESSGRAM scores.

## Results

### Participant characteristics (Table 1)

**Table 1:** Participant characteristics.

	Overall (n = 40)	Males (n = 23)	Females (n = 17)
Age (Years) (Mean (SD))	12.8 (1.9)	13.3 (1.7)	12.1 (2.0)
Weight (kg) (Mean (SD))	53.1 (12.7)	55.8 (13.0)	49.5 (11.8)
Height (cm) (Mean (SD))	159.7 (10.5)	163.7 (10.0)	154.3 (8.6)
BMI (Mean (SD))	20.6 (3.6)	20.6 (3.2)	20.7 (4.1)
BMI Z-Score (Mean (SD))	0.4 (1.0)	0.3 (0.9)	0.4 (1.2)
BMI Percentile (Mean (SD))	60.7 (28.4)	59.4 (28.1)	62.4 (29.4)
Obese (BMI > 95^th^ percentile) (n (%))	5 (12.5%)	1 (4.3%)	4 (23.5%)
V02 Max (ml∙kg^-1^∙min^-1^) (Mean (SD)):	43.5 (9.8)	47.0 (9.4)	38.8 (8.6)
V02 Max Fitness Categories (n (%))			
Very Poor	2 (5.0%)	2 (8.7%)	0 (0.0%)
Poor	8 (20.0%)	4 (17.4%)	4 (23.5%)
Fair	5 (12.5%)	3 (13.0%)	2 (11.8%)
Good	9 (22.5%)	6 (26.1%)	3 (17.6%)
Excellent	4 (10.0%)	4 (17.4%)	0 (0.0%)
Superior	12 (30.0%)	4 (17.4%)	8 (47.1%)
45-Second Squat Test Indices (Mean (SD)):			
Ruffier Index^1^	11.0 (4.1)	10.8 (4.2)	11.2 (4.2)
Dickson Index^2^	9.9 (4.3)	9.7 (4.3)	10.1 (4.4)
3-Minute Step Test Parameters (Mean (SD)):			
Step Ruffier^3^	15.4 (5.3)	14.4 (4.9)	16.7 (5.7)
Step Dickson^4^	14.5 (5.5)	13.2 (4.9)	16.3 (5.9)
Jacks Equation^5^	2.0 (0.3)	2.1 (0.3)	1.8 (0.4)
One minute post-Step Test Heart Rate	110.6 (26.0)	106.2 (24.6)	116.6 (27.3)
RPE (Mean (SD))			
Peak Ruffier	2.7 (1.4)	2.6 (1.3)	2.7 (1.6)
Peak Step Test	3.4 (1.7)	3.3 (1.7)	3.5 (1.6)
Peak VO2	6.8 (1.5)	7.1 (1.4)	6.2 (1.6)
Total PROMIS Score (Median (Q1, Q3))	26.0 (21.0, 31.5)	26.0 (21.0, 31.0)	26.0 (21.0, 32.0)
FITNESSGram Low Disease Risk (n (%))	26 (65.0%)	15 (65.2%)	11 (64.7%)

^1^ Ruffier index: (P0 + P1 + P2–200) / 10

^2^ Dickson index: ((P1–70) + 2(P2-P0))/10 (where P0 = heart rate (HR) at rest, P1 = HR immediately upon completion, P2 = HR after one minute of rest)

^3^ Step Ruffier: (T0 + T3 + T4–200) / 10

^4^ Step Dickson: ((T3–70) + 2(T4-T0))/10 (where T0 = resting HR before 3-minute step test, T3 = HR immediately upon completion, T4 = HR after one minute of rest)

^5^ Jacks VO2 Max = -2.045 + (height in inch*0.062) + 100*[1/3*(T1+T2+T3)/T0]*(-0.411) + (T0*0.011)

The mean age was 12.8 ± 1.9 years. The mean BMI was 20.6 ± 3.6 and mean BMI z- score was 0.4 ± 1.0. 12.5% of participants were obese (BMI > 95th percentile). Mean VO_2max_ was 43.5 ± 9.8 ml∙kg^-1^∙min^-1^. Most participants (30.0%) were superior in VO_2max_ fitness categories while 5.0% were very poor and 20.0% were poor. The median total PROMIS score was 26.0 and 65.0% of participants indicated FITNESSGram low disease risk.

### Feasibility and safety

No adverse events occurred during the testing and the burden to the participant was low. The staff was easily able to execute both the Squat and the Step Tests without complication. Thus, both were considered safe and feasible.

### Exercise test scores

**Fig 2:**
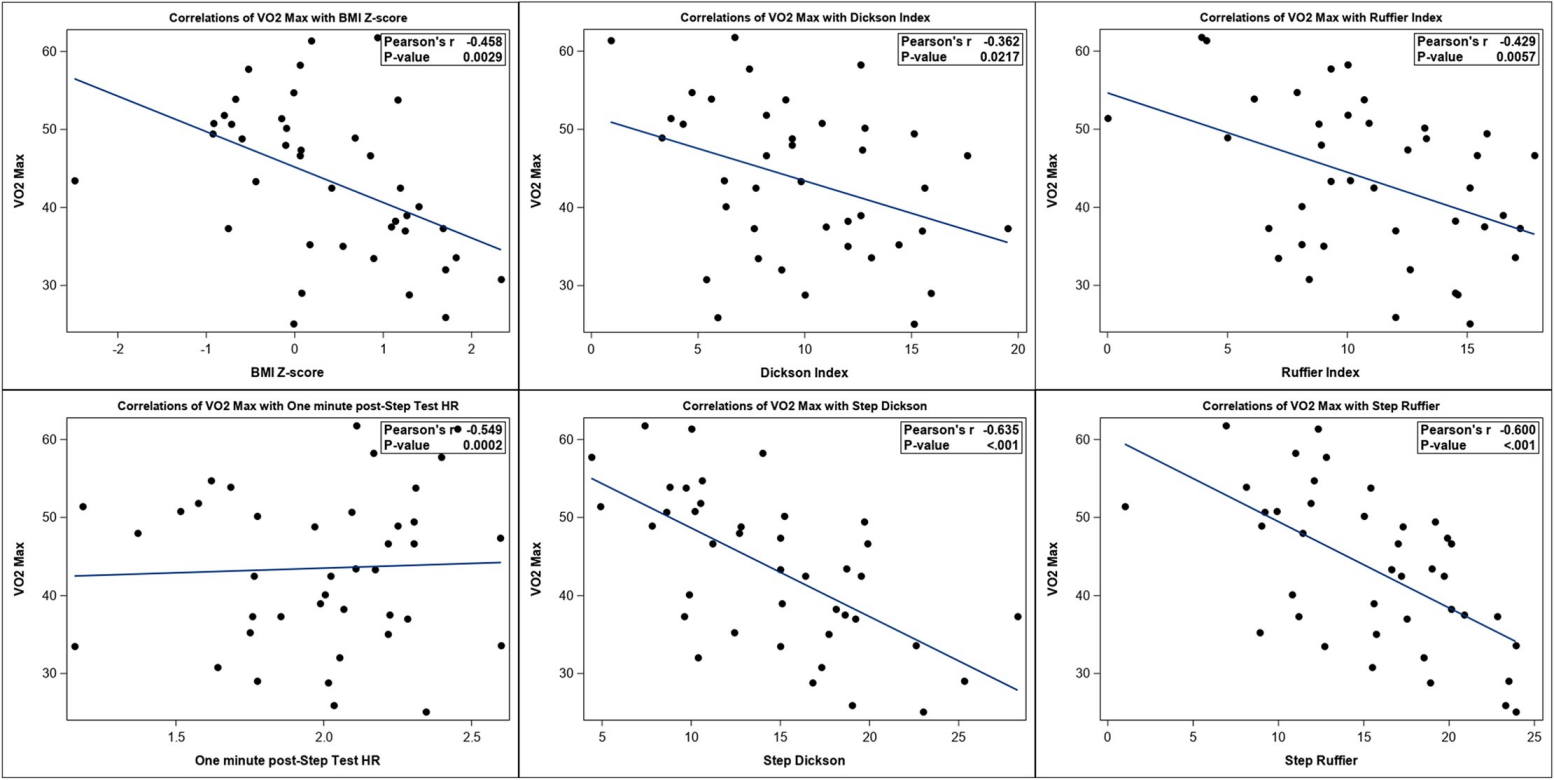
Correlations of VO_2max_ with BMI Z-score, Squat Test indices (Dickson and Ruffier index), and Step Test indices (one minute post-Step Test HR, Step Dickson, and Step Ruffier index). All were significantly correlated with p-values <0.05.

The Squat and Step Test scores were normally distributed and significantly correlated with the VO_2max_ scores in our patient population. Overall, the Step Test correlated best with VO_2max_. Pearson correlation coefficients were r = -0.429 (p = 0.0057) for Squat Ruffier /VO_2max_, r = -0.362 (p = 0.0217) for Squat Dickson/VO_2max_, and r = -0.549 (p = 0.0002) for one minute post-Step Test HR/VO_2max_ (**Table 2, Fig 2**). We further examined the correlation between VO_2max_ and Squat and Step Test by gender. Pearson correlation coefficients were r = -0.523 (p = 0.0104) for one minute post-Step Test HR/VO_2max_ in males, and r = -0.528 (p = 0.0294) for one minute post-Step Test HR/VO_2max_ in females. The Squat Test was significantly correlated with the VO_2max_ scores only in males but not in females (**Table 2**).

**Table 2:** Correlations of VO_2max_ with indices/scores.

		Overall		Males		Females	
	R^2^	r	p-value	r	p-value	r	p-value
Ruffier Index	0.184	-0.429	**0.0057**	-0.610	**0.0020**	-0.225	0.3854
Dickson Index	0.131	-0.362	**0.0217**	-0.510	**0.0129**	-0.191	0.4633
Topp Equation	0.002	0.043	0.7943	-0.035	0.8743	-0.267	0.3006
One minute post-Step Test HR	0.301	-0.549	**0.0002**	-0.523	**0.0104**	-0.528	**0.0294**
Step Ruffier	0.360	-0.600	**< .001**	-0.570	**0.0045**	-0.586	**0.0135**
Step Dickson	0.403	-0.635	**< .001**	-0.660	**0.0006**	-0.429	**0.0298**
BMI Z-score	0.210	-0.458	**0.0029**	-0.542	**0.0076**	-0.435	0.0806
Peak Ruffier	0.009	-0.095	0.5604	-0.076	0.7298	-0.106	0.6869
Peak Step Test	0.085	-0.292	0.0675	-0.304	0.1580	-0.305	0.2342
Peak VO_2_	0.065	0.254	0.1135	0.045	0.8386	0.299	0.2429
Total PROMIS Score	0.002	0.039	0.8107	-0.079	0.7212	0.215	0.4080

HR = Heart Rate

Bolded p-values denote statistical significance (p<0.05).

Ruffier index for Step Test and Dickson index for Step Test were correlated with the VO_2max_ overall and by gender but the Jacks Equation was not. The BMI z-score was also correlated with the VO_2max_ overall (r = -0.458, p = 0.0029) and in males (r = -0.542, p = 0.0076), but not in females. RPEs and PROMIS scores were not significantly correlated with VO_2max_ (**Table 2**).

### Discriminative ability of tests to find risk in children using FITNESSGRAM

The ROC AUC was poor for Squat Test Ruffier and Dickson indices (0.593 and 0.610, respectively). Compared to the Squat Test, the Step Test had better ability to discriminate risk in children using FITNESSGRAM with AUCs equal to 0.713 for the Step Test heart rate after one minute, 0.732 for Step Test Ruffier index, and 0.7280 for Step Test Dickson index. As expected, ability to discriminate was excellent for VO_2max_, (AUROC = 0.9505), though BMI z-score also had excellent performance (AUROC = 0.8132, **Table 3**).

**Table 3:** Discriminative ability using FITNESSGRAM.

	AUROC	95% Confidence Interval
Ruffier Index	0.5934	0.4095, 0.7774
Dickson Index	0.6099	0.4280, 0.7918
One minute post-Step Test HR	0.7129	0.3260, 0.7070
Step Ruffier	0.7321	0.5576, 0.9066
Step Dickson	0.7280	0.5685, 0.8876
BMI Z-score	0.8132	0.6742, 0.9522
VO_2max_	0.9505	0.8924, 1.0000

HR = Heart Rate.

## Discussion

This study examined the association between fitness scores from the VO_2max_ treadmill test, Squat Test and the Step Test and how well each test could discriminate low and high risk populations using FITNESSGram standards. Our main hypothesis was supported, as both the Squat and Step Test were correlated with the VO_2max_ treadmill test in this pediatric sample, and all three fitness determinations were able to discriminate low and high risk populations as defined by FITNESSGram standards [12]. The Step Test exhibited the strongest correlation with VO_2max_, and this correlation was observed for both males and females. In addition, the Step Test had the highest discriminative ability with respect to risk, and also had higher RPE scores, a potentially useful demonstrative tool in the pediatric clinic setting, where education around the value of vigorous exercise is important. These findings may be due to the longer duration of the Step Test as opposed to the Squat Test (3 minutes versus 45 seconds), allowing for heart rate increase and plateau. The Step Test one-minute post-test heart rate showed similar correlation to the VO_2max_ and similar discrimination of FITNESSGram scores as the more complicated calculated formulas, which may be beneficial when considering implementation in busy clinic settings.

To our knowledge, this is the first study to attempt to validate the Squat Test [13] relative to the VO_2max_ treadmill test in a pediatric age group. The Squat and Step Tests have been validated previously in adults [16, 27] and various submaximal step test protocols have been validated previously in children and adolescents [15, 28, 29]. However, this is the first study to show that all three fitness determinations may be able to discriminate low and high risk populations using FITNESSGram standards, an observation that can be used to estimate an individual patient’s risk and provide appropriate counseling within the clinic setting. As also reported in Hayes et al [28], our study found that BMI was correlated with VO_2max_. BMI was also able to effectively discriminate low and high risk populations using FITNESSGram standards in our patient population. As also seen in Tolusso et al [30], RPE scores did not correlate with VO_2max_ in our population. Limitations of our study included a small sample size, cross-sectional design, and a relatively lower BMI and potentially a selection bias with a more fit study population than the general population due to the nature of the study.

There are many potential benefits to monitoring cardiorespiratory fitness in pediatric clinics. Physical activity begins to decline in childhood and interventions should be targeted towards this population before their habits stabilize [31]. In addition, large health gains can be achieved by encouraging the most sedentary patients to increase their physical activity even modestly [4]. Simpler tests of cardiorespiratory fitness such as the Squat and Step Tests could add great value to assessment of cardiovascular risk in pediatric clinics. In our study, both tests were well-tolerated, easy to administer, and had no adverse events. The value of these tests above that of measuring a BMI z-score will need to be evaluated, since our study showed that BMI z-score discriminated low and high risk FITNESSGram scores with equal efficacy to that of these fitness determinations. Future directions will include implementation and study of these simple fitness tests longitudinally in the clinic setting.

## Abbreviations

AUROC: area under the receiver operating characteristic curve
BMI: body mass index
CRF: cardiorespiratory fitness
HR: heart rate
MPH: miles per hour
NIH: National Institutes of Health
PROMIS: NIH PROMIS Short Form Physical Activity Survey
RPE: Rate of Perceived Exertion Scale
VO_2max_: maximal rate of oxygen consumption
